# Relationship of the standard uptake value of ^18^F-FDG-PET-CT with tumor-infiltrating lymphocytes in breast tumors measuring ≥ 1 cm

**DOI:** 10.1038/s41598-021-91404-y

**Published:** 2021-06-08

**Authors:** Soeun Park, Eun-ki Min, Soong June Bae, Chihwan Cha, Dooreh Kim, Janghee Lee, Yoon Jin Cha, Sung Gwe Ahn, Joon Jeong

**Affiliations:** 1 Department of Surgery, Gangnam Severance Hospital, Yonsei University College of Medicine, 20, Eonju-ro 63-gil, Gangnam-gu, Seoul, 06229 Republic of Korea; 2 Department of Pathology, Gangnam Severance Hospital, Yonsei University College of Medicine, Seoul, South Korea; 3 Department of Surgery, CHA Ilsan Medical Center, CHA University, Goyang, South Korea; 4 Department of Surgery, Hanyang University Seoul Hospital, Hanyang University, Seoul, South Korea; 5 Department of Surgery, Hallym University Dongtan Sacred Heart Hospital, Hallym University College of Medicine, Hwaseong, South Korea

**Keywords:** Breast cancer, Cancer microenvironment

## Abstract

Evidence suggests that tumor cells and tumor-infiltrating lymphocytes (TILs) compete for glucose in the tumor microenvironment and that tumor metabolic parameters correlate with localized immune markers in several solid tumors. We investigated the relationship of the standardized uptake value (SUV) of ^18^F-fluorodeoxyglucose positron emission tomography computed tomography (^18^F-FDG-PET-CT) with stromal TIL levels in breast cancer. We included 202 patients who underwent preoperative ^18^F-FDG-PET-CT and had a tumor measuring ≥ 1 cm. Maximum SUV (SUVmax) was determined using ^18^F-FDG-PET-CT. Multiple logistic regression was used to identify factors related to high TIL levels (≥ 40%). All tumors were treatment naïve. A significant and weak correlation existed between continuous SUVmax and continuous TIL levels (p = 0.002, R = 0.215). Tumors with high SUVmax (≥ 4) had higher mean TIL levels than those with low SUVmax (< 4). In multivariable analysis, continuous SUVmax was an independent factor associated with high TIL levels; each 1-unit increment in SUVmax corresponded to an odds ratio of 1.14 (95% confidence interval: 1.01–1.29) for high TIL levels. Our study implies that SUV is associated with TILs in breast cancer and provides clinical evidence that elevated glucose uptake by breast tumors can predict the immune system-activated tumor micromilieu.

## Introduction

The tumor microenvironment (TME) is an ensemble of non-tumor cells including immune cells, fibroblasts, and endothelial cells^1^ and affects tumor development and progression through dynamic interactions with cancer cells^2^. Identification of TME interplays depending on the tumor’s metabolic activity has revealed that the TME regulates tumor metabolism^3^. A previous study has reported that the demand for glucose is increased in tumor cells and tumor-specific immune cells, and this competition for glucose uptake between tumor cells and immune cells can directly influence cancer progression^4^.

Tumor-infiltrating lymphocytes (TILs) are a cellular component of the immune system in the TME. Several clinical trials have reported the prognostic and predictive importance of TILs in breast cancer. In these trials, TIL levels have been highlighted as a biomarker for predicting treatment response to chemotherapy in patients with breast cancer^5–8^. The studies also found that tumors with high TIL levels have shown favorable prognosis among triple negative breast cancer (TNBC) or human epidermal growth factor receptor-2 (HER2)-positive breast cancer^7,9–12^. Furthermore, high TIL levels are associated with high-proliferative, high-grade, estrogen receptor (ER)-negative tumors and higher pathologic complete response (pCR) rates^8,13–17^.

^18^F-fluorodeoxyglucose-positron emission tomography-computed tomography (^18^F-FDG-PET-CT) provides important tumor-related qualitative and quantitative information on cancer based on glucose metabolism^18^. For breast cancer, a high maximum standardized uptake value (SUVmax) is related to tumor aggressiveness, advanced stage, and poor prognosis^19–23^.

To identify the relationship between tumor metabolic status and tumor immunogenicity, recent studies have reported the correlations between SUVmax and immune markers in gastric cancer, non-small cell lung cancer, and breast cancer^24–29^. In this study, we aimed to explore the correlation between the SUVmax of ^18^F-FDG-PET-CT and stromal TIL levels in breast cancer.

## Results

### Baseline characteristics

The characteristics of the 202 patients included in this study are summarized in Table 1. The median patient age was 53.2 years. Most tumors were invasive ductal carcinoma (86.1%) and showed favorable biology such as histological grade I or II (77.2%), nuclear grade 1 or 2 (66.3%), luminal/HER2(−) (71.8%), and low Ki-67 labeling index (L.I.) (55.0%). More than half of the tumors had a small tumor burden; 58.4% tumors had T stage 1 and 73.3% tumors were node-negative. The median SUVmax was 4.68, and the median TIL level was 15%.

**Table 1:** Patient characteristics.

	Number (%)
**Age (years), median (range)**	53.2 (24–87)
**Histology**	
Invasive ductal carcinoma	174 (86.1)
Invasive lobular carcinoma	17 (8.4)
Others	11 (5.4)
**T stage**	
1	118 (58.4)
2	80 (39.6)
3	4 (2.0)
**N stage**	
0	148 (73.3)
1	45 (22.3)
2	7 (3.5)
3	2 (1.0)
**Histologic grade**^1^	
I or II	156 (77.2)
III	45 (22.3)
**Nuclear grade**^1^	
1 or 2	135 (66.3)
3	65 (32.2)
**ER positive**	163 (80.7)
**PR positive**	137 (67.8)
**HER2 positive**^2^	42 (20.8)
**Subtypes**	
Luminal/HER2(−)	145 (71.8)
HER2(+)	32 (15.8)
TNBC	25 (12.4)
**Ki-67 labeling index**^1^	
< 14%	111 (55.2)
≥ 14%	90 (44.6)
**AR (%), mean (SD)**	87.01 (± 25.25)
**SUV max, median (range)**	4.68 (0.63–18.54)
**TIL (%), median (range)**	15 (5–95)

*ER* estrogen receptor, *PR* progesterone receptor, *HER2* human epidermal growth factor receptor-2, *TNBC* triple-negative breast cancer, *AR* androgen receptor, *SUV* standard uptake value, *TIL* tumor-infiltrating lymphocytes.

^1^Missing value.

^2^HER2 positivity was defined as 3 + on immunohistochemistry or amplification on fluorescence in situ hybridization.

### Tumor characteristics related to TIL levels

**Figure 1:**
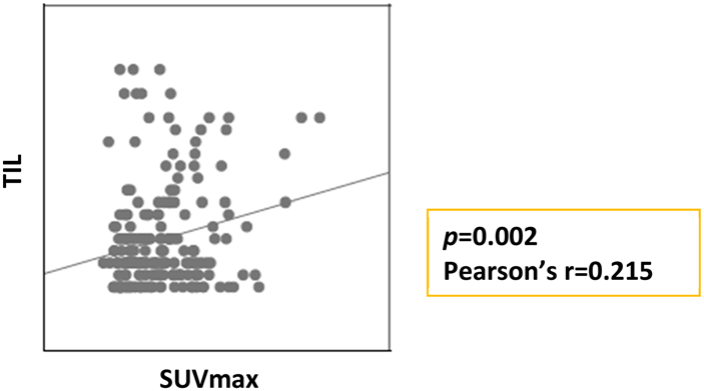
Correlation between the continuous maximum standardized uptake value (SUVmax) and tumor-infiltrating lymphocyte (TIL) levels.

We evaluated the correlation between continuous TIL levels and continuous SUVmax using Pearson’s correlation analysis (Fig. 1). There was a significant and weak correlation between continuous SUVmax and continuous TILs *(p* = 0.002, Pearson’s r = 0.215).

Next, we compared mean TILs according to tumor characteristics (Table 2). Invasive ductal carcinoma (IDC) showed significantly higher mean TIL levels than invasive lobular carcinoma or other histological cancer types. The mean TIL levels were higher in tumors with histological grade (HG) III than in those with HG I or II. Similarly, mean TIL levels were higher in tumors with nuclear grade (NG) 3 than in those with NG 1 or 2. Among the subtypes, luminal/HER2(-) tumors showed the lowest mean TIL levels. When divided by Ki-67 L.I. of 14%, tumors with high Ki-67 L.I. showed higher mean TIL levels than those with low Ki-67 L.I. We also compared mean TIL levels based on the categorized SUVmax with a cutoff value of 4^20,30^. Tumors with a high SUVmax value showed higher mean TIL levels than those with a low SUVmax value (*p* = 0.028, Mann–Whitney U test).

**Table 2:** Mean tumor-infiltrating lymphocyte levels (%) according to tumor characteristics.

	N	Mean (SD)	*p* value
**Histology**^1^			0.001
IDC	174	24.20 (± 21.98)	
ILC	17	11.27 (± 11.20)	
Others	11	13.64 (± 26.77)	
**T stage**^2^			0.426
1	118	21.03 (± 21.34)	
2–3	84	24.19 (± 22.65)	
**N stage**^2^			
0	148	23.41 (± 23.37)	0.536
1–3	54	19.85 (± 16.98)	
**Histologic grade**^2,3^			< 0.001
I or II	156	18.70 (± 19.68)	
III	45	35.74 (± 25.09)	
**Nuclear grade**^2,3^			< 0.001
1 or 2	136	17.54 (± 17.86)	
3	65	33.29 (± 25.99)	
**Subtypes**^1^			< 0.001
Luminal/HER2(−)	145	17.38 (± 15.90)	
HER2(+)	32	36.32 (± 27.97)	
TNBC	25	37.78 (± 29.58)	
**Ki-67 labeling index**^2,3^			0.003
< 14%	111	17.29 (± 17.45)	
≥ 14%	90	28.21 (± 24.29)	
**SUVmax**^2^			0.028
< 4	85	18.33 (± 21.25)	
≥ 4	117	25.33 (± 22.10)	

*IDC* invasive ductal carcinoma, *ILC* invasive lobular carcinoma, *HER2* human epidermal growth factor receptor-2, *TNBC* triple-negative breast cancer, *SUV* standard uptake value, *SD* standard deviation.

^1^Kruskal–Wallis test.

^2^Mann–Whitney U test.

^3^Missing value.

### Predictive factors for tumors with high TIL levels

We identified factors predicting TIL levels ≥ 40% using logistic regression analysis (Table 3). In univariable analysis, the significant variables were age, HG, NG, tumor subtypes, Ki-67 L.I., androgen receptor (AR) percentage, and continuous SUVmax. Because the tumor subtypes were decided based on a combination of estrogen receptor (ER), progesterone receptor (PR), and HER2 status, only tumor subtype was included in the multivariable model to avoid collinearity of variables. In multivariable analysis, tumor subtypes and continuous SUVmax remained an independent variable associated with high TIL levels, and NG was marginally significant (*p* = 0.050) for predicting high TIL levels. When the SUVmax increased by 1 unit, the probability of high TIL levels increased by 14% (95% confidence interval [CI]: 1.011–1.285, *p* = 0.032).

**Table 3:** Predictive factors for tumors with high tumor-infiltrating lymphocyte levels.

	Univariable analysis		Multivariable analysis	
	ORs (95% CIs)	*p* value	ORs (95% CIs)	*p* value
**Age**	1.029 (0.997–1.061)	**0.074**	1.017 (0.982–1.054)	0.337
**Histology**				
IDC	Ref	0.202		
ILC	0.209 (0.027–1.628)	0.135		
Others	0.335 (0.042–2.697)	0.304		
**T stage**				
1	Ref			
2–3	1.540 (0.778–3.048)	0.216		
**N stage**				
0	Ref			
1–3	0.824 (0.374–1.816)	0.631		
**Histologic grade**				
I or II	Ref			
III	4.873 (2.323–10.223)	** < 0.001**	1.508 (0.534–4.255)	0.438
**Nuclear grade**				
1 or 2	Ref			
3	4.375 (2.145–8.923)	** < 0.001**	**2.248** (**1.000**–**5.053**)	**0.050**
**ER( +)** (Ref: ( −))	0.234 (0.109–0.503)	** < 0.001**		
**PR( +)** (Ref: ( −))	0.258 (0.127–0.524)	** < 0.001**		
**HER2( +)** (Ref: ( −))	3.172 (1.496–6.722)	**0.003**		
**Subtypes**				
Luminal/HER2( −)	Ref	** < 0.001**		
HER2( +)	5.856 (2.472–13.873)	** < 0.001**	**3.166** (**1.218**–**8.229)**	**0.018**
TNBC	5.916 (2.316–15.112)	** < 0.001**	**3.706** (**1.329**–**10.340**)	**0.012**
**Ki-67 labeling index ≥ 14%** (Ref: <14%)	3.922 (1.863–8.258)	** < 0.001**	1.512 (0.604–3.785)	0.377
**AR (%)**	0.984 (0.972–9.995)	**0.005**	1.001 (0.983–1.020)	0.879
**SUVmax**	1.211 (1.092–1.344)	** < 0.001**	**1.140** (**1.011**–**1.285)**	**0.032**

Bold font is used to also highlight statistically significant values.

*ANC* absolute neutrophil count, *ALC* absolute lymphocyte count, *ER* estrogen receptor, *HER-2* human epidermal growth factor receptor-2, *IDC* invasive ductal carcinoma, *PR* progesterone receptor, *TNBC* triple-negative breast cancer, *OR* odds ratio, *CI* confidence interval, *AR* androgen receptor, *ILC* invasive lobular carcinoma.

**Figure 2:**
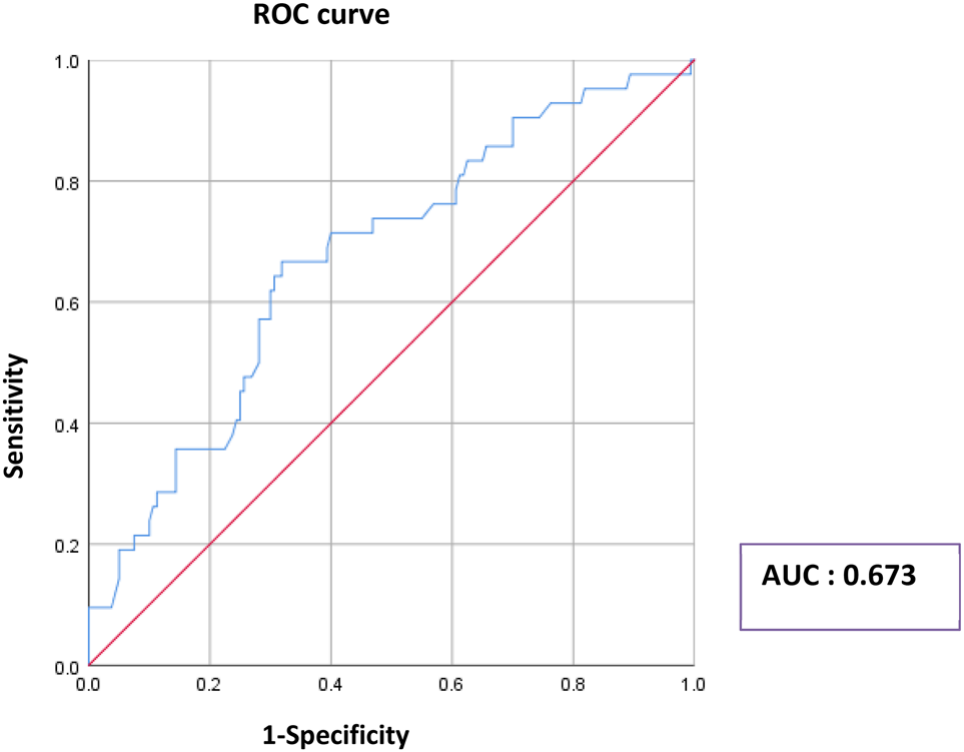
Area under the curve of the maximum standardized uptake value (SUVmax) predicting tumor-infiltrating lymphocyte (TIL) levels ≥ 40%

Additionally, to evaluate the predictive ability of continuous SUVmax for TIL levels ≥ 40%, we determined the area under the curve (AUC) using receiver operating characteristic (ROC) curves. The ROC curve for SUVmax in relation to TIL levels yielded an AUC of 0.673 (95% CI: 0.582–0.764, *p* = 0.001; Fig. 2).

## Discussion

In this study, we found an association between TIL levels and SUVmax in breast cancer (≥ 1 cm). Tumors with a high SUVmax value showed a higher mean TIL level than those with a low SUVmax value and there was a correlation between continuous SUVmax and TIL level. In multivariable analysis, SUVmax was an independent factor for predicting tumors with high TIL levels.

Correlations between SUVmax and TIL levels have been previously reported in non-small cell lung cancer and gastric cancer. In non-small cell lung cancer, SUVmax was correlated with CD8(+) TILs as well as CD163(+) tumor-associated macrophages, FoxP3(+) Treg cells, and PD-1(+) and PD-L1(+) cells^24^. In gastric cancer, SUVmax showed a marginal association with CD3(+) lymphocytes and a significant association with FoxP3(+) Treg cells^25^. In breast cancer, the relationship between SUVmax and TIL levels has been addressed in recent studies. Sasada et al. investigated this relationship using both whole-body positron emission tomography (PET) and dedicated breast PET (DbPET)^29^. SUVmax correlated with TILs in both whole-body PET and DbPET, and only DbPET was related to TIL levels after propensity score matching analysis. They suggested that DbPET could be more accurate in assessing fluorodeoxyglucose uptake in breast cancer, and their findings are in line with our finding that SUVmax is associated with TIL levels. Collectively, the abovementioned studies support our findings and suggest that SUVmax could have a potential role in assessing the immune system-activated tumor micromilieu.

In addition, these findings indicate that SUVmax can be a potential biomarker associated with immune-targeting therapy. In the study conducted by Hirakata et al.^26^, there were significant associations between SUVmax and PD-L1(+) TIL levels and between SUVmax and TIL levels. Since PD-L1 expression in immune cells has already been used in clinical practice to determine the use of atezolizumab as an anti-PD-L1-targeting monoclonal antibody in metastatic breast cancer^31,32^, it is worthwhile to address the predictive function of SUVmax in relation to the response of immune-check point inhibitors (ICIs).

Moreover, our previous genomic study revealed that Transforming growth factor (TGF) pathway genes, which attenuate the response to ICIs^33^, were significantly downregulated in tumors with a high SUV value^34^. Further, we found that the SUV signature was significantly associated with ICI responsiveness and improved overall survival in patients with urothelial cancer treated with ICIs. Taken together, our findings provide evidence that SUVmax can be assessed in terms of ICI responsiveness in breast cancer.

Our study has several limitations. First, this was a retrospective study conducted in a single institution, and there was a difference between patient characteristics, especially tumor subtypes. Because we excluded patients who received neoadjuvant chemotherapy, there were a small number of cases with more aggressive tumor types (HER2(+) or TNBC) and advanced disease. To overcome this, multivariable analysis was performed to demonstrate the independent capability of SUVmax in predicting high TIL levels. Second, although we used previously reported cutoff values for TIL levels and SUVmax, there are no standard cutoff values. Third, more specific immune biomarkers such as PD-L1 or the subpopulations of TILs were not assessed. A more detailed analysis is needed to comprehensively understand the mechanism underlying the relationship between TIL levels and SUVmax. Lastly, survival analysis was not performed due to the short follow-up period. Assessment of clinical outcomes could be helpful in identifying the prognostic capability of this relationship. Despite these shortcomings, our study has the advantage of enrolling the largest number of patients thus far.

In conclusion, we found associations between the SUVmax of ^18^F-FDG-PET-CT and stromal TIL levels in breast cancer. These results suggest that elevated glucose uptake in breast tumors can be used to predict the activation of the immune system in the TME. Further studies are warranted to comprehensively understand the interactions between the immune and metabolic systems in the TME and identify the clinical role of SUVmax of ^18^F-FDG-PET-CT in predicting TIL levels.

## Methods

### Patients

Between August 2016 and December 2017, we enrolled 202 patients with stage I-III breast cancer who underwent preoperative ^18^F-FDG-PET-CT followed by primary surgery at Gangnam Severance Hospital, Yonsei College of Medicine, South Korea. In these patients, we successfully evaluated the SUVmax values and stromal TIL levels. To circumvent the effect of chemotherapy on SUVmax values and TIL levels, patients who underwent preoperative chemotherapy were excluded. To address more clear relationship between SUVmax and stromal TIL levels by reducing the partial-volume effect of PET^35^, we included only patients with tumors measuring ≥ 1 cm.

Clinical data on age at the time of surgery, HG, NG, tumor size, ER status, PR status, HER2 status, AR percentage, and Ki-67 L.I. were collected from the medical database. Tumors were classified according to the tumor–node–metastasis staging of the American Joint Committee on Cancer, 7th edition, and tumor grade was determined using the modified Scarf-Bloomer-Richardson grading system^36^. The study was approved by the Institutional Review Board (IRB) of Gangnam Severance Hospital (Local IRB number: 2020-0950-001), which waived the requirement of informed consent due to the retrospective study design. The study was performed in accordance with good clinical practice guidelines and the Declaration of Helsinki.

### Assessment of TIL levels

TIL levels were measured as described in previous studies^37,38^. A pathologist (Y.J.C.) performed hematoxylin and eosin staining to review the histological features of treatment-naïve surgical specimens. Stromal TIL levels were evaluated according to the standardized methodology proposed by the international TIL Working Group^11^. The tumor area, defined by the presence of invasive tumor cells, was identified. All mononuclear cells including lymphocytes and plasma cells, but not polymorphonuclear leukocytes, were counted. The areas outside the tumor border, around the intraductal component, and around the normal lobules were excluded. Within the tumor border, areas showing crush artifacts and necrosis were also excluded. For each case, the average TIL level was measured using a representative section of the whole tumor, and the average level was reported as a percentage.

In this study, the cutoff value of high TIL level was set as 40%, which was used to analyze associations between TIL levels and pathological CR (pCR) or event-free survival in the NeoALTTO study^39^.

### ^18^F-FDG-PET-CT estimation

The procedure for ^18^F-FDG-PET-CT was the same as that previously reported^20^. After at least 8 h of fasting, patients received an intravenous injection of ^18^F-FDG (0.14 MBq) in the arm contralateral to the primary tumor. Sixty minutes after injection of ^18^F-FDG, whole-body positron emission tomography scans were obtained using a Philips Allegro PET camera (Philips Medical Systems, Cleveland, OH, USA). During the scans, patients were placed in the supine position with their arms raised. The SUV was calculated by measuring the ^18^F-FDG uptake by the primary tumor in the region of interest using the following formula: SUV = (maximal radioactivity concentration in the region of interest)/(injected dose/patient’s weight (kg)). The SUV cutoff value of 4 was determined according to previous studies^20,30,34^.

### Statistical analysis

Pearson’s correlation coefficient was calculated to measure the correlation between continuous TIL levels and SUV. According to tumor characteristics, mean TILs levels were compared using the Mann–Whitney U test or Kruskal–Wallis test. The Kolmogorov–Smirnov test was used to test the normal distribution of TIL data. The distributions of nonparametric variables were compared using the Mann–Whitney U test or Kruskal–Wallis test. The clinicopathological factors associated with high TIL levels (≥ 40%) were analyzed using logistic regression analysis. Variables that showed a significant difference (*p* < 0.10) in univariate analysis were entered in multivariable analysis. To evaluate the ability of continuous SUVmax to predict TIL levels ≥ 40%, we determined the AUC using ROC curves. All statistical tests were two tailed, and *p* < 0.05 was considered statistical significant. All statistical analyses were performed using SPSS, version 26.0 (SPSS, Chicago, IL, USA).

## Data Availability

The datasets generated during and/or analysed during the current study are available from the corresponding author on reasonable request.
